# Asking Young Adults about Their Attitudes toward and Knowledge of Sex in Later Life

**DOI:** 10.1093/geroni/igaa057.3220

**Published:** 2020-12-16

**Authors:** Iulia Fratila, Liza Berdychevsky

**Affiliations:** University of Illinois at Urbana-Champaign, Champaign, Illinois, United States

## Abstract

Sexual expression is a lifelong need related to health and wellbeing. However, older adults’ sexuality is often neglected and stigmatized due to societal ageist stereotypes portraying them as asexual. Although baby boomers’ generation resists such portrayals, societal acceptance of sexuality in later life is slow to materialize. The purpose of this study was to explore this acceptance among young adults while focusing on three research questions: (1) How much do young adults know about older adults’ sexuality and how do they feel about it? (2) Do young adults’ knowledge and views of later-life sexuality vary by gender? (3) Do young adults’ views of later-life sexuality vary based on their general attitudes toward sexuality? Data collection included online and intercept survey methods. The sample (N=270) was young (M=21.58 years, SD=4.32) and included 149 women and 113 men. Results revealed that young adults had medium levels of knowledge, yet rather permissive/open-minded attitudes regarding later-life sexuality. Higher levels of knowledge were unrelated to more permissive attitudes. Independent samples t-test revealed no differences by gender in young adults’ knowledge and attitudes. However, multiple regression results indicated that general liberal attitudes toward sexuality (β=.772, t=17.867, p=.000) and viewing sex as leisure activity (β=.147, t=3.338, p=.001) are significant predictors of having more permissive/open-minded attitudes toward older adults’ sexuality (R2=.557, F(3,266)=111.390, p=.000). These findings suggest that socio-psychological (rather than cognitive and demographic) factors drive the acceptance of later-life sexuality among young adults. This study offers valuable insights for knowledge, practice, and advocacy concerning older adults’ sexuality.

